# Assessment of Aerobic and Respiratory Growth in the *Lactobacillus casei* Group

**DOI:** 10.1371/journal.pone.0099189

**Published:** 2014-06-11

**Authors:** Teresa Zotta, Annamaria Ricciardi, Rocco G. Ianniello, Eugenio Parente, Anna Reale, Franca Rossi, Lucilla Iacumin, Giuseppe Comi, Raffaele Coppola

**Affiliations:** 1 Istituto di Scienze dell’Alimentazione-CNR, Avellino, Italy; 2 Scuola di Scienze Agrarie, Forestali, Alimentari e Ambientali, Università degli Studi della Basilicata, Potenza, Italy; 3 Dipartimento di Agricoltura, Ambiente e Alimenti, Università degli Studi del Molise, Campobasso, Italy; 4 Dipartimento di Scienze degli Alimenti, Università degli Studi di Udine, Udine, Italy; Université d’Auvergne Clermont 1, France

## Abstract

One hundred eighty four strains belonging to the species *Lactobacillus casei*, *L. paracasei* and *L. rhamnosus* were screened for their ability to grow under aerobic conditions, in media containing heme and menaquinone and/or compounds generating reactive oxygen species (ROS), in order to identify respiratory and oxygen-tolerant phenotypes. Most strains were able to cope with aerobic conditions and for many strains aerobic growth and heme or heme/menaquinone supplementation increased biomass production compared to anaerobic cultivation. Only four *L. casei* strains showed a catalase-like activity under anaerobic, aerobic and respiratory conditions and were able to survive in presence of H_2_O_2_ (1 mM). Almost all *L. casei* and *L. paracasei* strains tolerated menadione (0.2 mM) and most tolerated pyrogallol (50 mM), while *L. rhamnosus* was usually resistant only to the latter compound. This is the first study in which an extensive screening of oxygen and oxidative stress tolerance of members of the *L. casei* group has been carried out. Results allowed the selection of strains showing the typical traits of aerobic and respiratory metabolism (increased pH and biomass under aerobic or respiratory conditions) and unique oxidative stress response properties. Aerobic growth and respiration may confer technological and physiological advantages in the *L. casei* group and oxygen-tolerant phenotypes could be exploited in several food industry applications.

## Introduction

The *Lactobacillus casei* group includes three closely related species (*Lactobacillus casei*, *L. paracasei* and *L. rhamnosus*), involved in different food and health-related applications [1]–[3]. Their wide ecological distribution (human host, vegetable, meat and dairy products) and the potential role as probiotics makes these species interesting for the development of new functional foods and relevant for several genetic and physiological studies.

The taxonomy of the *L. casei* group is controversial and many studies have addressed the identification and genotypic characterization of strains belonging to the species *L. casei*, *L. paracasei* and *L. rhamnosus* [1], [4]. Recently, comparative genomic studies [5]–[7] highlighted the heterogeneity of *L. casei* group, suggesting that genome diversification contributes to ecological niche adaptation in these species. However, the presence of genes not always reflects their functionality and the physiological and technological properties of strains mainly depend on expressed features in a given set of conditions.

Like other lactic acid bacteria (LAB), *L. casei*, *L. paracasei* and *L. rhamnosus* are considered oxygen-tolerant anaerobes with fermentative metabolism, which normally lack both catalase and an active electron transport chain (ETC). The growth condition and the type of metabolism significantly affect the stress responses in LAB and, recently, several studies have demonstrated that conditions which promote aerobic and respiratory growth (presence of oxygen, heme and/or menaquinone in the substrate) induce in *Lactococcus lactis* and *L. plantarum* useful traits (improved biomass production and stress tolerance) for industrial and biotechnological applications [8], [9].

Supplementation with heme may promote the synthesis of a heme-dependent catalase and a bd-type cytochrome oxidase [8], [9]. Catalase protects cells against oxidative stress by degrading hydrogen peroxide (H_2_O_2_), while cytochrome bd oxidase, the final component of the minimal respiratory chain in LAB [8], [9], contributes to energy supply (through extra ATP generation) and depletion of intracellular oxygen. The biogenesis of catalase and cytochrome bd oxidase seems to be uncorrelated [10] and, in some cases, species lacking respiratory capability (*L. sakei*) encode a heme-dependent catalase (*kat*), while well characterised respiring species (*Lc. lactis*) lack the *kat* gene.

Menaquinone (vitamin K2), which acts as an electron shuttle in respiratory chain of LAB, is found in many vegetable and meat products and dietary intake may contribute to human health [11]. The ability to synthesize quinones varies among LAB: *Lc. lactis* subsp. *cremoris* MG1363, using the complete (mena)quinones biosynthesis complex *menFDXBEC*, produces and exploits menaquinone for respiratory growth, while other species (including *L. plantarum*) need exogenous menaquinone supplementation to perform respiration [9].

To date, with the exception of studies on *L. plantarum* [12]–[19], reports on the aerobic and/or respiratory metabolism in other species of *Lactobacillus* are rare [20]–[22]. Moreover, data on stress response mechanisms of *L. casei* group are limited to a small number of strains and conditions and, generally, have been carried out on cells grown in anaerobiosis.

*L. casei, L. paracasei* and *L. rhamnosus* are widely distributed in plant, animal and human-associated habitats, in which oxygen, heme and menaquinone may be present. Tolerance of oxygen and oxidative stresses may be important in the survival in different environments, including the gut, and during preservation of starter and probiotic cultures [8], [9]. In this work, we investigated the capability of a diverse collection of *L. casei*, *L. paracasei* and *L. rhamnosus* strains to cope with the presence of oxygen, ROS (reactive oxygen species) generating compounds, heme and menaquinone. The shift towards aerobic and respiratory growth has been considered, for the first time, in these species in order to identify respiration-competent strains and exploit the oxygen-tolerant phenotypes for development of improved starter and probiotic cultures.

## Materials and Methods

### Strains and Culture Conditions

One hundred eighty four strains belonging to the species *Lactobacillus casei*, *L. paracasei* and *L. rhamnosus*, isolated from different sources (Table 1), were used. All strains were identified to the species level using a polyphasic approach (SDS-PAGE, DGGE-PCR, specific-PCR, multiplex-PCR, High Resolution Melting Analysis) and genotyping was performed by RAPD-PCR, Rep-PCR, Sau-PCR (Iacumin et al. 2014, unpublished data).

**Table 1:** List of strains used in this study.

Source	Source group	Strains and species
Raw and heat-treated milk, yoghurt,milk machinery	Fresh dairy products (FD)	***L. paracasei***: LMG9192 *, LMG13087, P1E5, P1E6, P2P3, DSM5622
		***L. paracasei*** ** subsp. ** ***tolerans***: LMG9191
		***L. rhamnosus***: P1E4, HA111
Soft and hard cheeses (Scamorza, ParmigianoReggiano, Grana Padano, Spressa, Asiago,Montasio, Canestrato di Moliterno, Morlacco,Bellunese, Pecorino, Caciocavallo, Provolone,Emmenthal, Raclette de Savoie,chinese and tunisian cheeses)	Cheese (CH)	***L. casei***: LMG6904, CI4368
		***L. paracasei***: LMG25880, LMG25883, LMG12164, DBPZ291, DBPZ293, DBPZ0317, DBPZ0318, DBPZ420, DBPZ421, DBPZ422, DBPZ424, DBPZ434, DBPZ435, DBPZ450, DBP451, DBPZ472, DBPZ475, DBPZ476, DBPZ478, DBPZ635, DBPZ718, DBPZ733, DBPZ734, C4H8, M266, M268, M299, M308, M348, M354, M359, S1, S3, V3, W11, DSG03, DSG05, DSG07, ESG10, HSG09, PSG06, PSG09, PSG10, P71, TH1229, CF143, R61, F17, N24, H12, SP57, L24, TH406, TH1229, TMW1.1444, TMW1.1259
		***L. rhamnosus***: M15, O14, PRA204, PRA232, PRA331, DBPZ428, DBPZ430, DBPZ445, DBPZ446, DBPZ448, DBPZ449, FSG01, CI4362, CF1350, CF377, D44, H25, 5A9T, 5D9T, L9, L47, LACcas13, M307, M315
Fermented sausages	Meat products (M)	***L. paracasei***: CTC1675
		***L. rhamnosus***: CTC1676, 2220
Sourdoughs	Sourdoughs (SD)	***L. paracasei***: DBPZ561, DBPZ563, DBPZ564, DBPZ571, DBPZ572, DBPZ579, Q2, Q4, I1, I2, I3
Wine, wine wort, wine machinery	Wine (W)	***L. casei***: B166
		***L. paracasei***: LMG11961, LMG11963, LMG13717, LMG13731, B061, B161, B169, B171, B174, B195, B196, B350
		***L. rhamnosus***: B084, B170, B172, B173, B179
Beer, Elisir	Beverages (B)	***L. paracasei***: LCAcas25, LCAcas29, TMW 1.300
Corn step liquor; coffee	Plant material (P)	***L. casei***: DSM20178
		***L. rhamnosus***: DIAL40
Human (saliva, dental caries, blood,urethra, infant and adult faeces)	Human (H)	***L. casei***: LMG23516, N87, N811, N2014
		***L. paracasei***: LMG9438, LMG11459, LMG23511, LMG23518, LMG23523, LMG23538, LMG23543, LMG24098, LMG24101, LMG24132, DSM4905, DBTA34, N161, N42, N44, N76, N175
		***L. rhamnosus***: DBTA86, DBTC4, N1710, N171, N178, N715, N94, N95, N83,, N201, N209, N2012, N132, N22, N26, N812, N173, N1110, N131, N21, N172, N2010, N2013, N202, N25, N176, N2011, HA111, TMW 1.1538, DBPZ501, DSM20021, GG, R64
Unknown	Unknown (U)	***L. paracasei***: DBPZ525
		***L. rhamnosus***: DBPZ524

LMG: BCCM/LMG, Belgian Co-ordinated Collections of Micro-organisms (BCCM), Belgium.

DSM: DSM, Deutsche Sämmlung von Mikroorganismen und Zellkülturen, Braunschweig, Germany.

Other strains can be obtained from the Culture Collection of University of Basilicata.

*Strains which were used to evaluate the effect of heme and menaquinone supplementation (respiratory promoting conditions) on growth performances are underlined.

The strains were maintained as frozen and freeze-dried stocks in reconstituted 11% (w/v) Skim Milk containing 0.1% (w/v) ascorbic acid (RSM) in the culture collection of Scuola di Scienze Agrarie, Forestali, Alimentari e Ambientali, Università degli Studi della Basilicata, and routinely propagated (1% w/v) in MRS broth (Oxoid, Milan, Italy), pH 6.8 for 16 h at 37°C.

### Screening for Aerobic Growth and Catalase Production

All strains were screened in microplate (96-well) experiments for their ability to grow (16 and 42 h at 37°C) in a. anaerobiosis (AN; static cultivation in MRS broth in Generbox jars, bioMérieux SA, Marcy-l’Etoile, France, with AnaeroGen bags, Oxoid), b. aerobiosis (AE; in MRS broth, agitation on a rotary shaker at 150 rpm; Unimax 2010, Heidolph Instruments GmbH & Co.KG, Germany) and c. heme-supplemented aerobiosis (AEH; in MRS broth with 2.5 µg/mL hemin, initial pH 6.8, agitation on a rotary shaker at 150 rpm). Microplates (180 µL substrate/well) were inoculated with 20 µL of standardized (OD_450_ = 2.0) overnight anaerobic MRS-pre-cultures. Optical density at 450 nm (OD_450_; Titertek Multiskan Plus 311 BO Microplate Reader) and pH values (Double Pore Slim electrode, Hamilton Company, Reno, Nevada, USA) were measured at 16 h and 42 h on two replicates.

Production of catalase was qualitatively assayed by re-suspending the washed biomass (final OD_650_ = 1.0) derived from 1 mL of AN, AE and AEH cultures (16 h, 37°C) in 100 µL of 3% (v/v) H_2_O_2_. Bubble formation provided an indication of the presence of catalase activity in cell suspensions [23].

### Tolerance of Oxidative Stresses

A preliminary test was performed to select the concentration of H_2_O_2_ or ROS generators (menadione or pyrogallol) which provided the best discrimination between sensitive and tolerant strains. Ten strains (final OD_450_ = 2.0) were randomly chosen and cultivated (inoculum 10% v/v, 16 h at 37°C in microplates) in MRS with 0.16 g/L bromocresol purple (MRS-BCP), pH 6.8, containing H_2_O_2_ (ten two-fold dilutions from 880 to 1.7 mM) or pyrogallol (ten two-fold dilutions from 200 to 0.4 mM) or spotted (5 µL) on MRS agar plates containing menadione (9 two-fold dilutions from 0.4 to 0.0015 mM). Change of colour from purple to yellow (H_2_O_2_ and pyrogallol) or spot development (menadione) was considered as positive results. Appropriate concentrations of H_2_O_2_ (1 and 2 mM), pyrogallol (25 and 50 mM), menadione (0.15 and 0.2 mM) were selected and all strains were exposed to oxidative stresses as described above.

### Assessment of Respiratory Growth in Selected Strains

The effect of menaquinone supplementation was further evaluated in 60 strains (shown in boldface in Table 1) selected on the basis of stress response properties (heat, acid, osmotic, bile; Reale et al. 2014, unpublished data) and capability to tolerate oxygen and ROS (this study). WMB medium [17] was used to ensure the absence of heme (i.e. meat extract in MRS) during anaerobic and unsupplemented aerobic growth and a lower glucose concentration (10 g/L instead of 20 g/L; WMB10) was used to reduce the effect of carbon catabolite repression, if any, on the shift towards aerobic and respiratory metabolism [12]. Cultivation was carried out in 24-well microplates in: a. anaerobiosis (AN), b. aerobiosis (AE) and c. respiratory promoting condition (RS; AE cultivation in presence of 2.5 µg/mL hemin and 1 µg/mL menaquinone). Microplates (1 mL substrate/well) were inoculated with 20 µL of standardized (final OD_650_ = 1.0) WMB-pre-cultures and incubated at 37°C for 16 and 24 h. All trials were run in duplicate and increases of OD_650_ (SmartSpec Plus Spectrophotometer, Bio-Rad Laboratories) and pH values (Double Pore Slim electrode, Hamilton Company, Reno, Nevada, USA) were measured after 16 and 24 h. The oxygen consumption by AN, AE and RS cells was measured as described by Ricciardi et al. [24]. Briefly, cells were recovered by centrifugation and re-suspended (OD_650_ = 1.0) in air-saturated PB7 (potassium phosphate buffer, 20 mM, pH 7.0) containing 5.5 mM glucose and 0.002 g/L of resazurin (redox indicator) and the time of discolouration (h) from blue oxidized form (resazurin) to colourless reduced form (dihydroresofurin) was used as indicator of oxygen uptake.

### Effect of Aerobic and Respiratory Growth on Catalase Activity and H_2_O_2_ Tolerance in *L. casei*

Since only 4 strains of *L. casei* (CI4368 from cheese and N87, N811, N2014 from human faeces; Table 1) tolerated 2 mM of H_2_O_2_ and showed catalase-like activity, a further test was performed to confirm the presence of enzymatic activity and evaluated the effect of oxygen and heme/menaquinone supplementation on tolerance of H_2_O_2_.

AN, AE and RS cell suspensions (final OD_650_ = 1.0) were exposed (30 min, 37°C) to serial dilutions of H_2_O_2_ (ten two-fold dilutions from 880 to 1.7 mM). The survivors (if any) were cultivated in microplates as described before. Change of colour from purple to yellow and turbidity were considered as indication of the presence of survivors. Catalase-like activity was measured on the AN, AE and RS cell free extracts (obtained by mechanical lysis in FastPrep-24 Instrument, MP Biomedicals, Santa Ana, California, USA; 5 cycles of 60 sec at speed 6.0) according to the modified protocol of Risse et al. [27]. Briefly, AN, AE and RS samples were first incubated (15 min, 37°C) with 16 mM H_2_O_2_ (final concentration) and successively (10 min, 37°C) with a mixture containing 4-amino-antipyrine (3 mmol/L), sodium 3,5-dichloro-2 hydroxybenzenesulfonate (10 mmol/L) and peroxidase (0.28 U/mL). The residual amounts of H_2_O_2_ were spectrophotometrically measured at 510 nm. One µkatal (µkat) was defined as the amount of enzyme required to degrade 1 µmol H_2_O_2_/s. All measurements were run in duplicate.

### Effect of Aerobic and Respiratory Conditions on the Kinetics of Growth of Selected *L. paracasei* Strains

Five *L. paracasei* strains, showing different behaviours in response to aerobic and respiratory conditions (SP57, optimal growth in AE at both 16 and 24 h; R61, optimal growth in AE only at 16 h; B061, optimal growth in RS at both 16 and 24 h; C4H8, optimal growth in RS only at both 16 h; V3, oxygen-sensitive anaerobe; Table 1), were selected for further kinetic growth studies. All strains were re-cultivated at 37°C in: a. anaerobiosis (AN, screw-cap tubes filled with WMB10 containing 0.1 M MOPS, buffered WMB10, initial pH 6.8), b. aerobiosis (AE; 250 mL baffled shaking flasks with 50 mL buffered WMB10, agitation on a rotary shaker, 150 rpm) and c. respiration (RS, AE growth in presence of 2.5 µg/mL hemin and 1 µg/mL menaquinone). Anaerobic pre-cultures were used as inocula (5 log cfu/mL). Samples were aseptically withdrawn (30 min-interval) until to the late exponential growth phase (10 h) and after 24 h of incubation. Viable counts were performed on WMA (WMB pH 6.8 with 1.2% w/v agar), using a Whitley Automated Spiral Plater 2 (WASP2; Don Whitley Scientific Limited, UK), and colonies were enumerated with the EasyCount 2 colony counter (bioMérieux) after 48 h of incubation at 37°C in anaerobisosis. Lag time and maximum specific growth rates (µ_max_) were estimated with the primary biphasic model of Baranyi and Roberts [25] using the DMFit v 2.0 program [26].

H_2_O_2_ in supernatants and catalase activity in cell free extracts (obtained by mechanical lysis as described above) were measured as described by Risse et al. [27]. The activities of enzymes related to the oxygen metabolism (pyruvate oxidase, POX; NADH oxidase, NOX; NADH peroxidase, NPR) were measured as described by Quatravaux et al. [13]. Protein concentration was measured using the Bradford method [28]. Oxygen uptake by AN, AE and RS cells was evaluated using the resazurin assay [24]. All growth experiments and analytical measurements were run in duplicate.

### Comparative in Silico Analysis of the Genes Involved in Oxygen Utilization, Respiratory ET Chain and ROS Degradation

Genomes and gene sequences used for the comparative *in silico* analysis were retrieved from both Integrated Microbial Genomes platform (IMG; http://img.jgi.doe.gov./cgi-bin/w/main.cgi) and Gene section of NCBI database (http://www.ncbi.nlm.nih.gov). Gene sequences from *L. plantarum* WCFS1 (*pox3* and *pox5*, enconding for pyruvate oxidases; *nox5*, NADH oxidase; *npr2*, NADH peroxidase; *cydABCD*, synthesis and transport of cytochromes; *ubiE*, ubiquinone/menaquinone biosynthesis methyltransferase; *kat*, heme-dependent catalase), *L. plantarum* ATCC14431 (*MnKat*, manganese-dependent pseudocatalase), *L. sakei* subsp. *sakei* 23K (*sodA*, superoxide dismutase) and *Lc. lactis* subsp. *cremoris* MG1363 (*menFDXBEC*, complete menaquinone biosynthesis complex) were used as query to search homologous within the finished genomes of *L. casei* (ATCC334, BD-II, BL23, LC2W, Zhang) and *L. rhamnosus* (GG, GG-ATCC53103, K-ATCC8530, Lc705) as well as in the permanent draft genome sequences of *L. paracasei* 8700∶2 and ATCC25302.

Unidirectional (genes vs genomes) sequence similarity was detected using the IMG tools modifying, for each selected gene and genome, the BLAST cut-offs parameters (E-value, minimum % of identity).

### Statistical Analyses

Statistical (analyses of variance, correlations, two-way contingency tables) and graphic analyses were performed using SYSTAT 13.0 for Windows (Systat Software Inc., Richmond, CA, USA), while the Matrix Hierarchical Cluster Analysis (normalized data, Pearson distance, Average linkage UPGMA method) was obtained with PermutMatrix program v. 1.9.3 (LIRMM, France).

## Results

### Heterogeneity of *L. casei* Group in the Aerobic Growth and ROS Tolerance

**Figure 1:**
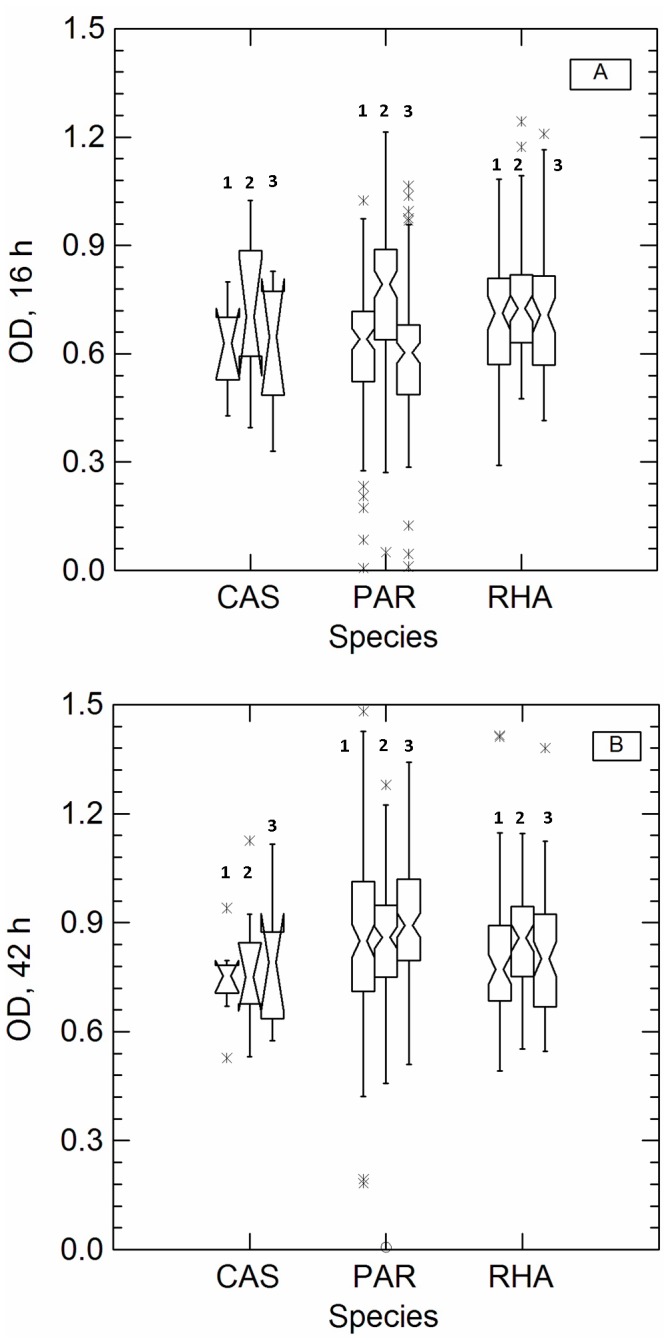
Notched box and whiskers plots showing the distribution of increase in optical density at 450(OD) after 16 (panel A) and 42 (panel B) h of cultivation in anaerobiosis (AN, box 1), aerobiosis (AE, box 2) and heme-supplemented aerobiosis (AEH, box 3). Species: CAS, *L. casei*; PAR, *L. paracasei*; RHA, *L. rhamnosus*. The notches indicate the median and its confidence limits; whiskers indicate range of data within ±1.5 interquartile range; x symbols indicate outlier strains.

All strains of *L. casei* group were able to cope with aerobic conditions, even if a large variability in growth behaviour was found (Figures 1a and 1b). For many (about 70%) strains (mostly belonging to the species *L. casei* and *L. rhamnosus*) the presence of oxygen and heme-supplementation enhanced growth compared to anaerobic cultivation, while for some *L. paracasei* strains aerobiosis and heme apparently impaired growth. Heterogeneity in OD and pH values, between anaerobic and aerobic conditions, was less noticeable after 42 h of incubation (data not shown).

All strains grown in heme-supplemented aerobiosis were also tested for their tolerance of ROS generating compounds to evaluate if aerobic growth (adaptation to oxygen and activation of antioxidant enzymes) and heme (synthesis of heme-dependent catalase and cytochrome bd oxidase) improved tolerance of oxidative stresses.

With the exception of 4 *L. casei* strains (CI4368, N87, N811, N2014), which surprisingly showed a catalase-like activity and survived to 2 mM of H_2_O_2_, none of the strains tolerated even the lowest (1 mM) H_2_O_2_ concentration used in this study. All strains (except LMG6904 and LC3) of *L. casei* and the 16% of *L. paracasei* survived to 0.2 mM menadione, while only 5 strains *L. rhamnosus* were tolerant. Most strains (100% of *L. casei*, 97% of *L. rhamnosus* and 76% of *L. paracasei*), instead, tolerated well the exposure to 50 mM pyrogallol (Table 2). Oxidative stress tolerance was not significantly associated to the isolation source.

**Table 2:** Frequency of strains tolerant of H_2_O_2_, menadione and pyrogallol.

		Tolerance of ROS generating compounds					
		H_2_O_2_ (mM)		Menadione (mM)		Pyrogallol (mM)	
		1	2	0.15	0.20	25	50
**Species** a	**Total** b	**(%)** c		**(%)** c		**(%)** c	
CAS	8	50	50	77	75	100	100
PAR	109	0	0	29	16	89	76
RHA	67	0	0	7	3	100	97
**Sources** a	**Total** b						
B	3	0	0	0	0	100	33
D	91	1	1	18	9	91	82
H	53	6	6	20	17	97	90
M	3	0	0	0	0	100	100
P	2	0	0	50	50	100	100
SD	11	0	0	54	36	73	64
U	2	0	0	0	0	100	100
W	19	0	0	16	16	100	95

a Species: CAS, *L. casei*; PAR, *L. paracasei*; RHA, *L. rhamnosus*. Sources: B, beverages; D, dairy products; H, human sources; M, meat products; P, plant material; SD, sourdoughs; U, unknown; W, wine.

b Total number of isolates.

c Frequencies (%) of tolerant strains, calculated using the two-way contingency table.

### Assessment of Respiratory Growth in Selected Strains of *L. casei* Group

The effect of menaquinone and, thus, the activation of a possible respiratory pathway, were further evaluated on the growth 60 selected strains.

**Figure 2:**
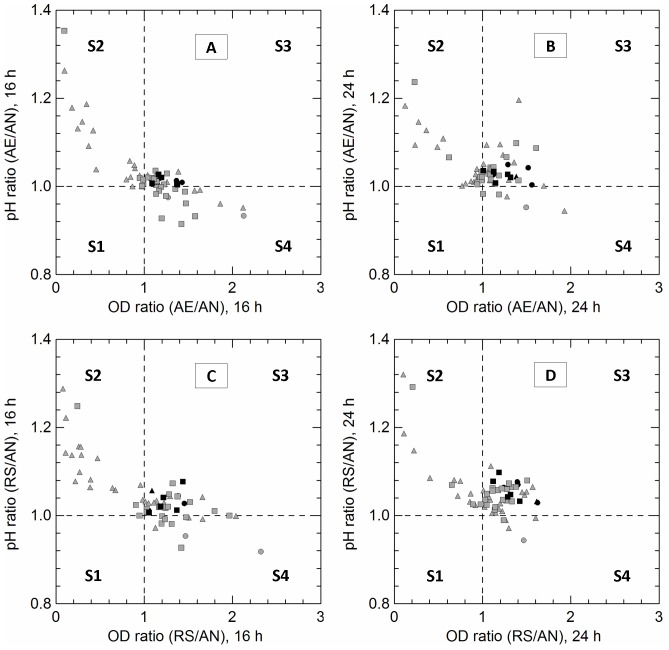
Distribution of *L. casei* (circles), *L. paracasei* (triangles) and *L. rhamnosus* (squares) strains on the basis of OD_650__nm_ (OD ratio AE/AN; OD ratio RS/AN) and pH (pH ratio AE/AN; pH ratio RS/AN) ratios. AN, anaerobiosis; AE, aerobiosis; RS, respiration. Graphs were divided in 4 sections by dashed lines to better represent the strains whose growth was stimulated (OD and pH ratios higher than 1) by aerobiosis (panel **A** and **B**, respectively at 16 and 24 h) or respiration (panel **C** and **D,** respectively at 16 and 24 h). Black symbols show the strains capable to consume oxygen (measurement with resazurin assay).

Growth and acid production in anaerobiosis, unsupplemented aerobiosis (AE) and respiration (RS) are compared in Figures 2a (AE, 16 h), 2b (AE, 24 h), 2c (RS, 16 h) and 2d (RS, 24 h). Ratios between optical density (OD) and pH values measured in AE, RS and AN cultivations were calculated to identify oxygen-tolerant and respiration-competent phenotypes. Several strains (section S3 of the graphs) exhibited a concurrent increase of OD_650_ and pH values (OD and pH ratios >1; these are the common traits of aerobic and respiratory growth; 20) when cultivated in presence of air (AE/AN ratios) or air and cofactors (RS/AN ratios), and some of them (14 strains) were able to consume oxygen in some or all conditions (black symbols in the section S3 of the graph). A smaller number of isolates (9 strains) grew better only under anaerobic conditions (section S2 of the graphs).

**Figure 3:**
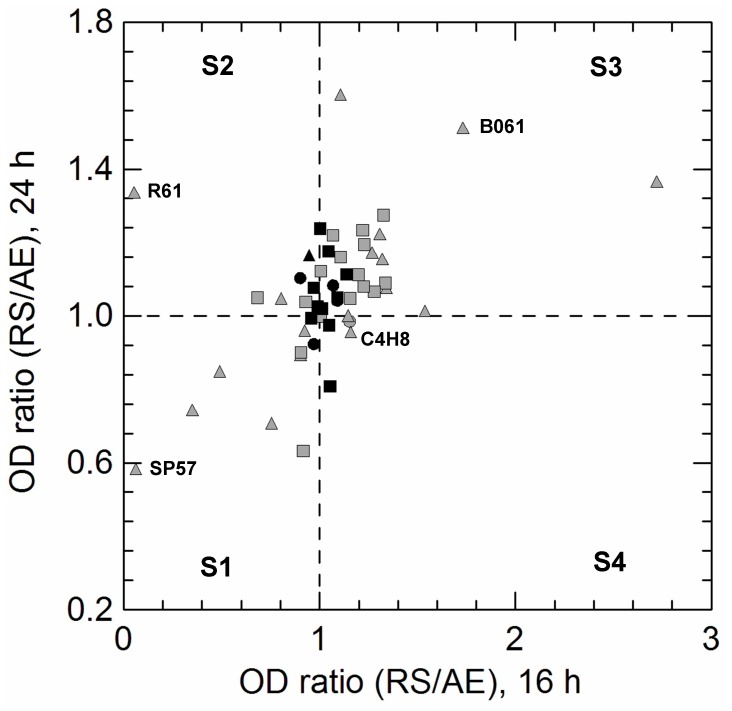
Distribution of *L. casei* (circles), *L. paracasei* (triangles) and *L. rhamnosus* (squares) strains on the basis of biomass production at 16 h (X-axis) and 24 h (Y-axis) under aerobic (AE) and respiratory (RS) growth. Graphs were divided in 4 sections by dashed lines to better represent the strains whose growth was stimulated (OD ratios higher than 1) by heme and menaquinone supplementation. Black symbols show the strains capable to consume oxygen (measurement with resazurin assay). The labels for strains which were used for growth kinetic studies are shown.

The effect of heme/menaquinone supplementation on growth (OD ratio between RS and AE conditions at 16 h and 24 h) of 51 oxygen-tolerant strains was also evaluated (Figure 3). The presence of respiratory cofactors generally improved growth compared to unsupplemented aerobiosis (Figure 3, section S3), but for a few strains seemed to impair growth (Figure 3, section S1).

The capability to consume oxygen in LAB may be related to activity of flavin-dependent oxidases (pyruvate oxidase, POX; NADH oxidase, NOX), which may be produced under both AE and RS condition, or to the activity cytochrome oxidase in respiratory ET chain (RS growth only). The time of resazurin discoloration (as indication of oxygen uptake) was shorter in presence of heme and menaquinone (ratio RS/AE of discoloration time <1, at both 16 h and 24 h,), suggesting a boost of oxygen uptake by the cytochrome oxidase activity in respiratory cells. The reduction of resazurin measured at 16 h (late exponential phase) and 24 h (stationary phase) was uncorrelated in both aerobic (r = 0.282) and respiratory (r = 0.556) cells, suggesting that the activity of enzymes involved in the oxygen utilization may be related with the growth phase and physiological state of cells.

### Classification of Strains on the Basis of Anaerobic, Aerobic and Respiratory Growth Pattern

**Figure 4:**
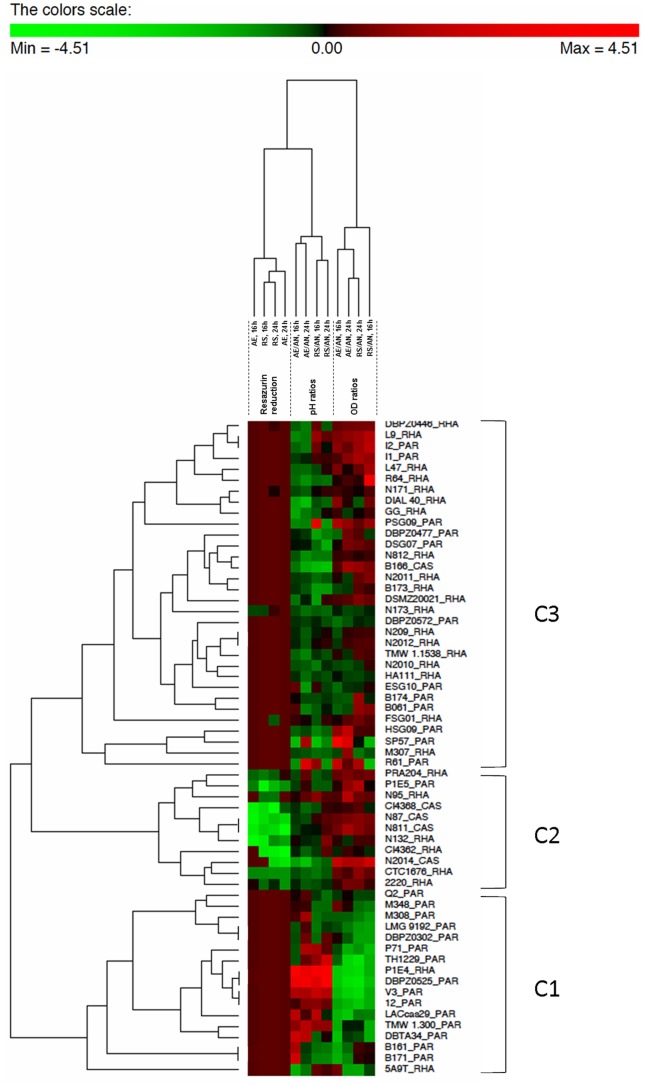
Matrix Hierarchical Cluster Analysis obtained with PermutMatrix program v. 1.9.3 (LIRMM, France). z-transformation of the dataset, Pearson r correlations and Average linkage (UPGMA) method were used to perform clustering. Column dendrogram: time of resazurin reduction in aerobic and respiratory cells after 16 and 24 h of incubation (columns 1, 2, 3, 4); pH ratios >1, AE/AN and RS/AN, after 16 h and 24 h of incubation (columns 5, 6, 7, 8); OD ratios >1, AE/AN and RS/AN, after 16 h and 24 h of incubation (columns 9, 10, 11, 12). Row dendrogram: strain_species. Colour scale: from green (negative data; minimum value is −4.51) to red (positive data, maximum values is +4.51), indicates the change from the mean in standard deviation units.

To correlate the parameters of aerobic and respiratory growth (increases of biomass and pH, oxygen uptake), a Matrix Hierarchical Cluster Analysis (MHCA; Figure 4) was performed on the 60 selected strains using as variables the rate of resazurin reduction and the concurrent increase of biomass and pH values (OD and pH ratios AE/AN or RS/AN >1) measured in anaerobic, aerobic and respiratory cells, at both 16 and 24 h. A z-value transformation was used for all variables.

Classification generated 3 major clusters that allowed to distinguish the strains in: oxygen-sensitive anaerobes (cluster C1; exclusively *L. paracasei*), unable to consume oxygen and for which aerobic and respiratory conditions impaired growth compared to anaerobic cultivation; respiration-competent strains (cluster C2; exclusively *L. casei* and *L. rhamnosus*), with oxygen uptake and increased biomass production in both AE and RS and pH in RS conditions resulting, possibly, by the shift towards aerobic and respiratory pathways; a large heterogeneous group (cluster C3) including oxygen-tolerant anaerobes, with increased growth under AE and or RS conditions, but with limited oxygen consumption ability.

### Effect of Aerobic and Respiratory Growth on Catalase Activity and H_2_O_2_ Tolerance in *L. casei*

Oxygen and supplementation with heme and menaquinone affected catalase activity and tolerance of H_2_O_2_ in *L. casei*. The four strains of *L. casei* that showed catalase-like activity and robustness to H_2_O_2_ in the screening step were further investigated to confirm the nature (pseudo- or heme-dependent catalase) of the enzyme and the H_2_O_2_ tolerance. The strains were able to degrade H_2_O_2_ in both anaerobic (AN, from 12.0 to 13.4 µmol H_2_O_2_/sec/mg protein) and aerobic (AE, from 13.0 to 14.8 µmol H_2_O_2_/sec/mg protein; RS, from 14.6 to 25.0 µmol H_2_O_2_/sec/mg protein) conditions, suggesting that the enzyme could be constitutive. Respiratory growth resulted in increased catalase activity, and improved survival to H_2_O_2_. In fact all strains survived up to 25 mM H_2_O_2_ when cultivated in AN and AE conditions and up to 100 mM when cultivated under RS conditions.

### Investigation of Factors Affecting the Lowest Adaptation to Aerobic Growth in *L. paracasei*

**Figure 5:**
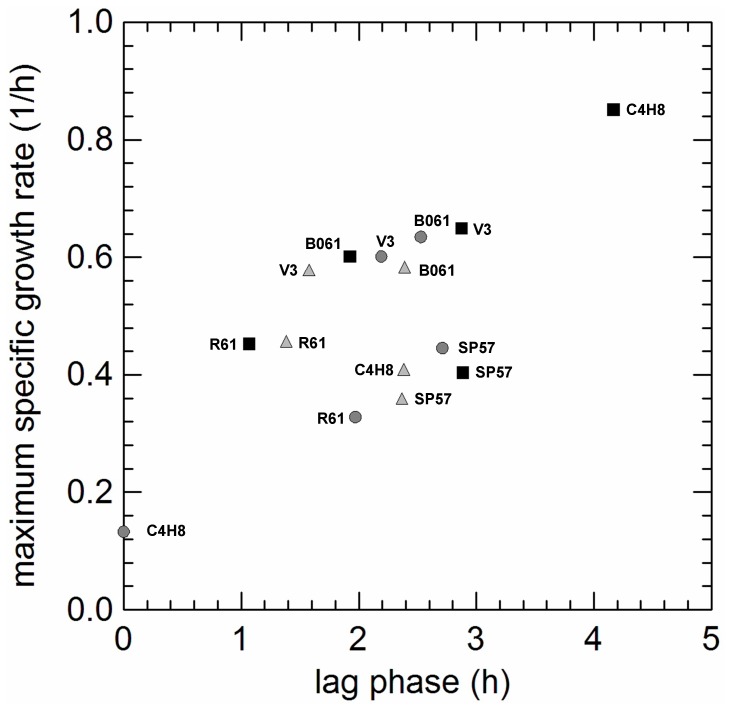
Relationships between maximum specific growth rates (Y-axis) and lag phases (X-axis) estimated in 5 *L. paracasei* strains (B061, C4H8, R61, SP57, V3) cultivated in anaerobiosis (light grey circles), aerobiosis (grey triangles) and respiratory (black squares) promoting condition.

Since *L. paracasei* exhibited the lowest adaptation to the aerobic growth (Figure 3, grey triangles; Figure 4, cluster C1 and C3), we investigated the duration of lag phase, the µ_max_ values, the activities of catalase, POX, NOX and NPR, the production of H_2_O_2_ and the capability to consume oxygen in 5 strains showing different phenotypes when cultivated in AN, AE and RS conditions. The biphasic model of Baranyi and Roberts [25] provided an excellent fit for all growth curves (R^2^ from 0.975 to 0.998). Anaerobic inocula were used for all cultivations and, thus, the time of adaptation mainly depended on the presence of oxygen, heme and menaquinone. Tolerance of oxygen and utilization of respiratory cofactors differed among strains. Supplementation and, to a lesser extent, aerobiosis significantly increasing lag phase of strain C4H8, while no lag phase was observed in AN cultivation), but reduced the lag phase in strains R61 and B061. Heme and menaquinone delayed the entry in exponential phase for the strains SP57 and V3, while unsupplemented aerobiosis seemed to offer a net gain in their growth, compared to AN cultivation (Figure 5).

Catalase, POX, NOX, NPR and oxygen consumption were undetectable at 10 h of incubation, suggesting that the maximum specific growth rate (µ_max_) and the cell number at the end of exponential growth phase were affected by oxygen and heme/menaquinone inhibition, rather than by the production of enzymes related to the aerobic metabolism. Cell numbers measured at the end of exponential phase were significantly correlated to growth rates (r = 0.967) but, surprisingly, the lag phase and µ_max_ were positively correlated for some strains (C4H8 and V3; a longer adaptation period positively affected the growth rate) or completely uncorrelated for other (SP57, R61 and B061, Figure 5).

**Figure 6:**
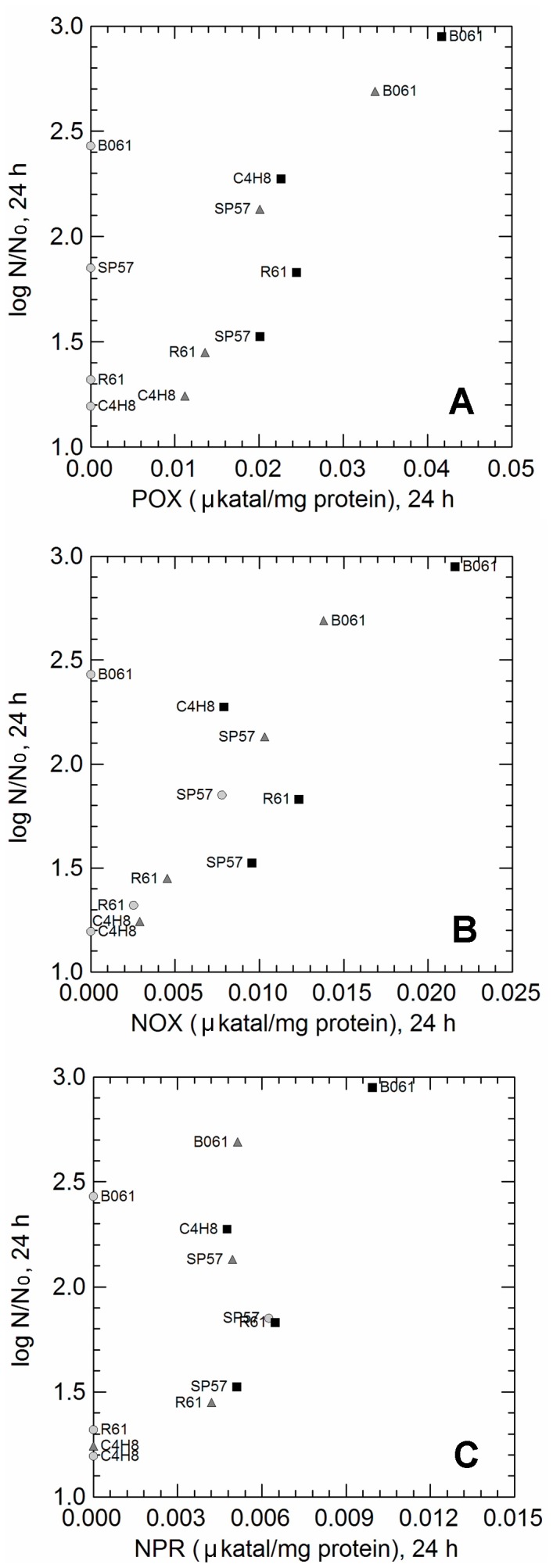
Correlations between cell numbers (log N/N_0_) reached up 24 h of incubation (Y-axis) and activities (µkatal/mg protein; X-axis) of oxygen-related enzymes (pyruvate oxidase, POX, panel A; NADH oxidase, NOX, panel B; NADH peroxidase, NPR, panel C) measured in 5 *L. paracasei* strains (B061, C4H8, R61, SP57, V3) cultivated in anaerobiosis (light grey circles), aerobiosis (grey triangles) and respiratory (black squares) promoting condition.

Contrarily to catalase (which was never detected), POX, NOX and NPR were detected after 24 h of cultivation. POX activity was found only in aerobic and respiratory growing cells (Figure 6a), suggesting that the enzyme is strictly related to aerobic growth. The presence of POX, that leads the conversion of pyruvate into acetate by POX-acetate kinase (ACK) pathway, was also confirmed by the increased pH in aerobic and respiratory cultures. Strain V3, which grew better only in anaerobiosis, was discarded from Figure 6 (a, b, c) because it was unable to synthesise any of the flavin-depend oxidases, which may explain its oxygen-sensitive phenotype. Cell numbers at the end of incubation (24 h) were significantly (*p* value <0.05) correlated with the activities of POX (r = 0.694), NOX (r = 0.710) and NPR (r = 0.614) (Figure 6a, b, c) and, with exception of strain SP57 for which heme and menaquinone supplementation had a negative effect on growth, increased in aerobiosis and even more in heme and menqauinone supplenented aerobiosis, when significant levels of POX, NOX, NPR were measured. Although no catalase activity was found, H_2_O_2_ was undetectable in aerobic/respiratory supernatants, probably because of degradation by NPR. Despite the presence of flavin-dependent oxidases, no singificant oxygen consumption was observed, suggesting that the strains were able to tolerate oxygen and inactivate ROS, but were unable to use it as a final electron acceptor in the respiratory chain (all strains belonged to cluster C3 in Figure 4).

## Discussion

This work highlights the capability of strains belong to the *L. casei* group to shift towards aerobic and respiratory metabolism in the presence of oxygen, heme or heme/menaquinone supplementation. For several strains, oxygen and respiratory cofactors enhanced growth performances compared to anaerobic cultivation. Brooijmans et al. [20] first revealed in *L. rhamnosus* (strain B637) the typical traits (increased biomass, due to extra ATP generation, and final pH, due to the conversion of pyruvate into acetate by POX-ACK pathway) of aerobic and respiratory growth and searched the *L. casei* ATCC 334 genome (currently the type strain for *L. paracasei*; [29]) for the presence of *cyd*-genes complex. These observations were limited to single strains, while here we tested the distribution of aerobic and respiratory phenotypes in a large number of isolates.

We found that *L. casei* and *L. rhamnosus* exhibited the best adaptation to the aerobic conditions and most strains were also capable to consume oxygen. Respiratory phenotypes were widespread among human isolates, and some cheese strains had showed evidence of aerobic metabolism and tolerated oxidative stress, while these traits were less frequent among strains isolated from other foods/environments thus confirming niche specific traits in *L. casei* and *L. rhamnosus* [5], [7].

The phenotypic data found in our screening study are supported by genomic information. Sequence comparisons (http://img.jgi.doe.gov./cgi-bin/w/main.cgi; http://www.ncbi.nlm.nih.gov), in fact, revealed in *L. casei*, *L. paracasei* and *L. rhamnosus* the presence of genes having homology with *pox5*, *nox5*, *npr2* (minimum % of identity from 40 to 50%) and the cytochrome gene-set *cydABCD* (minimum % of identity from 50 to 60%) of *L. plantarum* WCFS1. The complete (mena)quinones biosynthesis complex *menFDXBEC* of *Lc. lactis* subsp. *cremoris* MG1363 was absent in all species of the *L. casei* group, even if a methylase for ubiquinone/menaquinone biosynthesis (*ubiE* in *L. plantarum* WCFS1 and *Lc. lactis* subsp. *cremoris* MG1363) was annotated in the available genomes of *L. casei* and *L. rhamnosus* (40% minimum identity).

We also found that in some *L. paracasei* strains heme supplementation impaired the growth of aerobic cultures, suggesting a reduced ability to use this compound and/or toxic accumulation into the cells. The regulation of both heme efflux and transport systems (required to maintain homeostasis and to avoid the damage of free heme accumulation) has been recently investigated in respiring *Lc. lactis* MG1363 cells [30]–[32], but remains unclear in *Lactobacillus* species and may be among the factors contributing to the poor adaptation of *L. paracasei* to aerobic conditions. Additionally, as demonstrated by kinetic studies on 5 selected *L. paracasei* strains, lag phase and µ_max_ were differently affected by aerobic growth and supplements and the adaptation to aerobic and/or respiratory condition was strain-specific. In the stationary phase, instead, several metabolic pathways (heme utilization, activation of antioxidant enzymes, ROS degradation, extra energy supply by POX and/or cytochrome oxidase activities) may occur and affect positively the biomass production in oxygen-tolerant phenotypes. Heterogeneity in oxygen tolerance has already been demonstrated in several strains of *L. plantarum* group [40].

When cultivated in presence of oxygen, LAB can produce toxic reactive species (ROS; including hydrogen peroxide H_2_O_2_, superoxide anion O_2_^−^ and hydroxyl radical HO·) [32]. Most LAB have developed defence systems mainly based on the synthesis of antioxidant enzymes, such as flavin oxidases, superoxide dismutase, catalase, and peroxidases. The production of a manganese-dependent superoxide dismutase (MnSOD) has been demonstrated in several *Streptococcus* [33], [34], *Lactococcus* [35] and *Enterococcus* [36] strains, while the presence of a heme-dependent catalase (Kat) has been extensively studied in *L. sakei* [23], [37], [38] and *L. plantarum* [39], [40]. The ability to produce a manganese-containing catalase (heme-independent pseudocatalase; MnKat) by *L. plantarum* ATCC14431 has been also reported [41], [42].

Available genomes sequences of *L. casei* group do not include genes annotated as either heme-dependent catalase or Mn-dependent pseudocatalase. *L. casei* and *L. paracasei*, contrarily to *L. rhamnosus*, have sequences encoding for superoxide dismutase (50% of minimum identity with the *sodA* gene of *L. sakei* subsp. *sakei* 23K; http://img.jgi.doe.gov./cgi-bin/w/main.cgi; http://www.ncbi.nlm.nih.gov).

Recently, recombinant approaches have been used to express *sod* and *kat* in *L. rhamnosus* [38], [32] and catalase in *L. casei* [37], [43] to improve their oxidative stress tolerance.

In this study almost all strains were highly sensitive to H_2_O_2_ and the isolates of *L. rhamnosus* were more sensitive than *L. casei* and *L. paracasei* to the superoxide stress, thus confirming genomic information. However, we unexpectedly found that four *L. casei* strains were able to degrade H_2_O_2_ in all growth conditions, indicating the presence of a putative catalase-like enzyme. Levels of enzymatic activity were significantly higher than those reached by *L. casei* mutants of Rochat et al. [37] and Wang et al. [43] and the degradation of H_2_O_2_ was also observed under anaerobic cultivation without heme supplementation. This suggests that in our strains catalase might be constitutive and not oxygen- or heme-inducible as in other LAB [39]. Heme supplementation was not required for catalase synthesis but improve the amounts of catalase and robustness to H_2_O_2_ in this strains,but the reasons for this are not clear.

Several authors have demonstrated by comparative genomic analysis [7], [44], [45] that the acquisition of exogenous genes or gene clusters in *L. casei* can occur through horizontal gene transfer (HGT) and this mechanism may improve the fitness of strains and explain their genetic and metabolic versatility. Acquisition of genes encoding for catalase-like activity in our *L. casei* strains could be due to the transfer of genetic elements by other microorganisms sharing the same ecological niches and could contribute to the adaptation of *L. casei* to different habitats. Production of H_2_O_2_ and ROS compounds may be harmful for gut mucosal cells, contributing to the increase of inflammatory bowel diseases (IBD; [46]), and, therefore, the identification of *L. casei* strains with intrinsic H_2_O_2_- and ROS-degrading activities could be relevant in human health studies. Investigation on the evolutionary pathway of these strains and characterization and sequencing of this enzyme are in progress.

While we focused on the enzymes directly implicated in H_2_O_2_ and ROS degrading activity (catalase, NADH peroxidase, superoxide dismutase), other mechanisms may be involved in the oxidative stress tolerance and free radical degradation in the *L. casei* group. Genome analysis (http://img.jgi.doe.gov./cgi-bin/w/main.cgi; http://www.ncbi.nlm.nih.gov), in fact, reveals the presence of sequences encoding for thioredoxin (*trxA*)-thioredoxin reductase (*trxB*) system, leading of intracellular thiol/disulfide balance. Serata et al. [47] recently investigated the role of *trxA-trxB* on the growth and survival of *L. casei* Shirota under aerobic conditions, demonstrating its implication in oxygen and H_2_O_2_ tolerance. The *trxA-trxB* system could be implicated in the capability to cope with aerobic conditions and oxidative stresses even in strains which are unable to activate an aerobic metabolism and its role in *L. casei*, *L. rhamnosus* and *L. paracasei* needs further investigations.

This study highlighted the different behaviour and capability of *L. casei*, *L. paracasei* and *L. rhamnosus* in aerobic and respiratory metabolism, and demonstrated that *L. paracasei* has the lowest adaptation to aerobic growth among the *L. casei* group. Our results, moreover, revealed that aerobic and respiratory pathway may confer several physiological (increased biomass and oxidative stress response, synthesis of antioxidant enzymes) and metabolic (increase of external pH, extra energy production, prevention of oxygen accumulation) advantages and further studies need to be undertaken to exploit the oxygen-tolerant phenotypes for the development of competitive starter and probiotic cultures for use in food (improving the organoleptic properties following the reduction of H_2_O_2_) and health (degradation of anti-nutritional and toxic compounds; potential implication in common and degenerative diseases) related applications.

Aerobic bacteria, in addition to a complete respiratory ET chain, also possess a full and functional oxidative tricarboxylic acid (TCA) cycle. LAB have an interrupted or incomplete citric acid cycle (KEGG pathway; http://www.genome.jp/kegg/). Pedersen et al. [9] have classified LAB species in (a) respiring activated by heme supplementation, (b) respiring activated by heme and menaquinone supplementation and (c) non-proficient for respiration, based on the capability to activate a stripped-down respiratory chain. We feel that our data support a more flexible classification of LAB based on growth, metabolic and oxidative stress behaviours under aerobic conditions in (a) oxygen-sensitive anaerobes, (b) oxygen-tolerant anaerobes and (c) respiration-competent or defective aerobes.
